# Effect of Hydrogen Sulfide on Essential Functions of Polymorphonuclear Leukocytes

**DOI:** 10.3390/toxins15030198

**Published:** 2023-03-04

**Authors:** Sarah Farahat, Salome Kherkheulidze, Stephan Nopp, Alexander Kainz, Margherita Borriello, Alessandra F. Perna, Gerald Cohen

**Affiliations:** 1 Department of Nephrology and Dialysis, Medical University of Vienna, A-1090 Vienna, Austria; 2 Department of Precision Medicine, University of Campania “Luigi Vanvitelli”, 80138 Naples, Italy; 3 Department of Translational Medical Science, University of Campania “Luigi Vanvitelli”, 80131 Naples, Italy

**Keywords:** hydrogen sulfide, polymorphonuclear leukocytes, apoptosis, signal transduction, immunology, inflammation, chronic kidney disease, uremic toxins

## Abstract

Impaired polymorphonuclear leukocyte (PMNL) functions contribute to increased infections and cardiovascular diseases in chronic kidney disease (CKD). Uremic toxins reduce hydrogen sulfide (H_2_S) levels and the anti-oxidant and anti-inflammatory effects of H_2_S. Its biosynthesis occurs as a side process of transsulfuration and in the disposal of adenosylhomocysteine, a transmethylation inhibitor and proposed uremic toxin. PMNL chemotaxis was measured by the under-agarose method, phagocytosis, and oxidative burst by flow cytometry in whole blood and apoptosis by determining DNA content by flow cytometry and morphological features by fluorescence microscopy. Sodium hydrogen sulfide (NaHS), diallyl trisulphide (DATS) and diallyl disulphide (DADS), cysteine, and GYY4137 were used as H_2_S-producing substances. Increased H_2_S concentrations did not affect chemotaxis and phagocytosis. NaHS primed PMNL oxidative burst activated by phorbol 12-myristate 13-acetate (PMA) or *E. coli*. Both DATS and cysteine significantly decreased *E. coli*-activated oxidative burst but had no effect on PMA stimulation. While NaHS, DADS, and cysteine attenuated PMNL apoptosis, GYY4137 decreased their viability. Experiments with signal transduction inhibitors suggest that the intrinsic apoptosis pathway is mainly involved in GYY4137-induced PMNL apoptosis and that GYY4137 and cysteine target signaling downstream of phosphoinositide 3-kinase.

## 1. Introduction

Hydrogen sulfide (H_2_S) inhibits essential enzymes and exerts toxic effects at several levels [1,2]. Therefore, H_2_S was previously only known as a dangerous gas. However, recent studies have shown that H_2_S can also be produced endogenously, thus exhibiting important physiological effects at lower concentrations [3]. For example, H_2_S is able to reduce the production of pro-inflammatory cytokines, chemokines, and enzymes [4] and is involved in the regulation of blood pressure [5]. By regulating the activity of endothelial cells, vascular smooth muscle cells, and perivascular nerves, H_2_S leads to vasorelaxation [6]. Recently, it has also been shown that H_2_S mediates plaque stability [7]. H_2_S has both neuro-modulating and neuro-protective functions by increasing the production of the anti-oxidative glutathione in neurons exposed to oxidative stress [8,9].

Along with the more familiar carbon monoxide and nitric oxide, H_2_S belongs to the family of gasotransmitters. Under physiological conditions, it is mostly dissociated (86%), and only 14% exists as a gas. Gaseous H_2_S can easily diffuse through cell membranes due to its lipophilicity [1].

The plasma levels of H_2_S in patients with coronary heart disease, hypertension, and smokers are lower compared to the healthy reference group [5]. Chronic kidney disease (CKD) is also associated with significant reduction of H_2_S plasma levels [5]. Deficiency of H_2_S and its anti-oxidant, anti-inflammatory, and cytoprotective properties may contribute to the progression of CKD and mortality [2]. H_2_S biosynthesis inhibitors significantly worsen renal parameters [10].

The retention of many substances that are normally filtered by healthy kidneys leads to the development of the uremic syndrome. Retention solutes that negatively interact with biologic functions are called uremic toxins [11] and play a central role in impairing polymorphonuclear leukocyte (PMNL) functions in CKD patients [12]. Uremic toxins are responsible for the reduction in H_2_S concentration and its anti-oxidant and anti-inflammatory effects. While H_2_S is decreased in uremic patients on hemodialysis, lanthionine, a non-proteinogenic amino acid and byproduct of H_2_S biosynthesis, is increased [13]. Due to its ability to impair H_2_S synthesis in hepatoma cells, it is considered a novel uremic toxin [14]. Furthermore, the uremic toxin indoxyl sulfate impairs H_2_S formation in renal tubular cells [15]. Cyanate, a uremic toxin produced by the decomposition of urea in CKD patients, leads to a carbamoylation reaction [16,17,18] that abolishes the antioxidant and cytoprotective activity of H_2_S [19].

Immune dysfunction and particularly disturbed PMNL functions are a main cause of the high risk of infections and cardiovascular diseases and, as a result, increased morbidity and mortality in patients with CKD [12]. PMNLs are cells of the primary non-specific cellular immune response and play a crucial role in the defense against bacterial and fungal infections. Disturbed functions of PMNLs lead to a higher risk of bacterial infections and cardiovascular diseases and are a major cause of the increased risk of morbidity and mortality among CKD patients [12]. After chemotactic movement to the source of infection, PMNLs take up the invading microorganisms by phagocytosis and kill them with proteolytic enzymes intracellularly released from granula and with reactive oxygen species (ROS) produced during the oxidative burst. These critical PMNL functions can be impaired, leading to infectious diseases or pre-activated/primed, causing inflammation and subsequently cardiovascular disease. The coordinated elimination of activated PMNLs is essential for the resolution of inflammation [20]. Increased apoptosis results in a decreased immune response, whereas delayed apoptosis of PMNLs or impaired clearance of apoptotic PMNLs by macrophages causes inflammation [21]. In uremia, the balance between pro- and anti-inflammatory and between pro- and anti-apoptotic factors is disturbed.

In this study, we investigated the in vitro effect of H_2_S on several essential PMNL functions using the H_2_S-producing substances sodium hydrosulfide (NaHS), diallyl trisulfide (DATS) and diallyl disulfide (DADS), cysteine, and GYY4137.

## 2. Results

### 2.1. Chemotaxis

**Figure 1:**
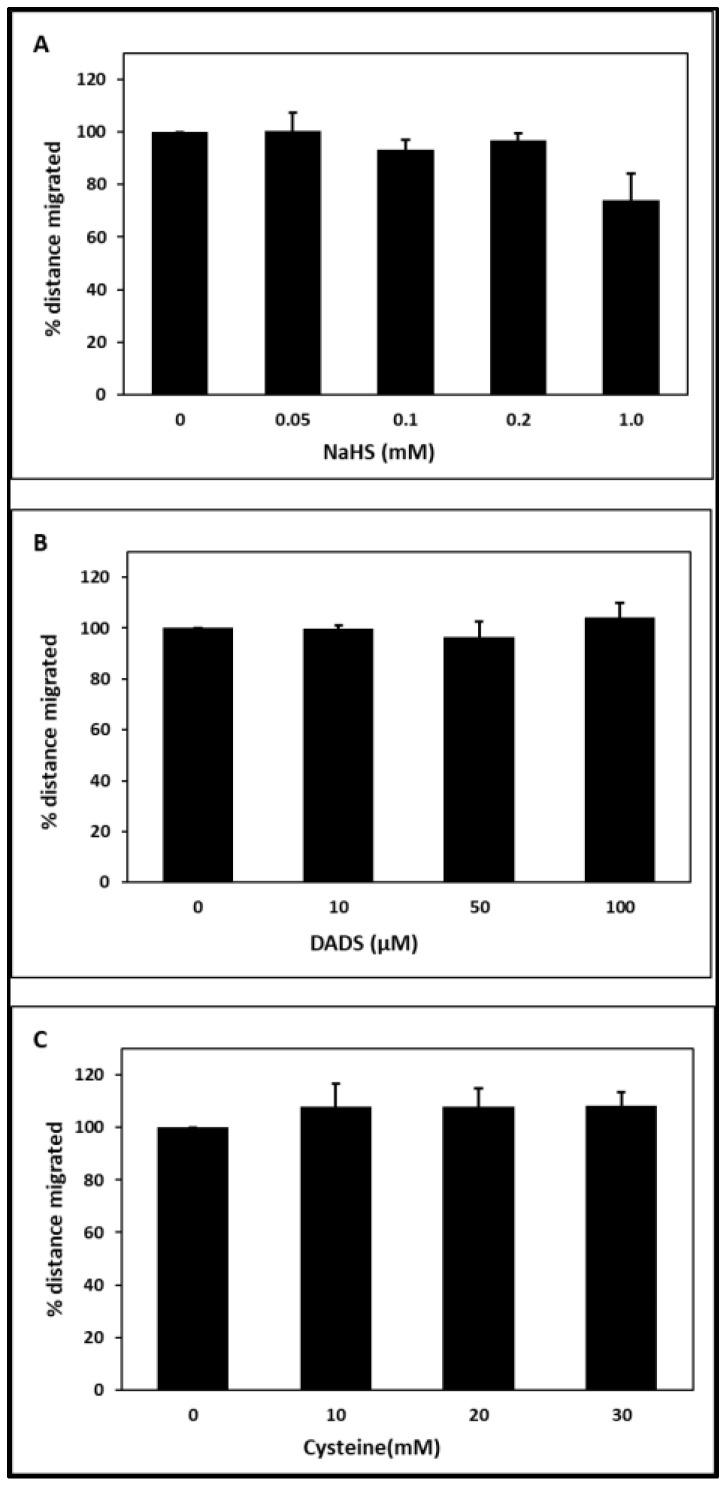
Effect of the H_2_S-releasing substances NaHS (**A**), DADS (**B**), and cysteine (**C**) on PMNL chemotaxis. The distance migrated in the absence of substance was set as 100%. n = 4–10 for A; n = 5 for B; n = 4–6 for C. NaHS: sodium hydrosulfide, DADS: diallyl disulphide.

Figure 1 shows the concentration dependency of the H_2_S-releasing substances NaHS, DADS, and cysteine on the chemotactic movement of PMNLs. None of the substances had a significant effect under the experimental conditions. The decrease of chemotaxis in the presence of the highest concentration of NaHS did not reach statistical significance.

### 2.2. Phagocytosis

**Figure 2:**
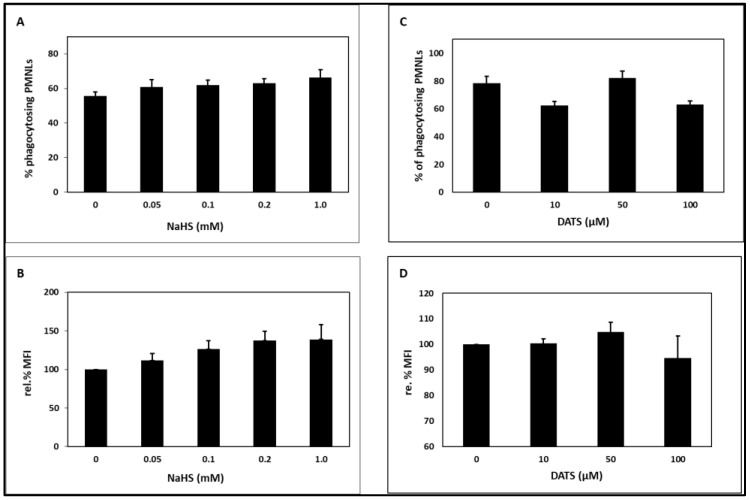
Effect of the H_2_S-releasing substances NaHS (**A**,**B**) and DATS (**C**,**D**) on PMNL phagocytosis. (**A**,**C**): percentage of PMNLs taking up *E. coli*; (**B**,**D**): relative percentage MFI as a measure of the number of *E. coli* cells taken up per PMNL. The value in the absence of substance was set as 100%. n = 3–7 for (**A**,**B**); n = 3–6 for (**C**,**D**). NaHS: sodium hydrosulfide, DATS: diallyl trisulfide, MFI: mean fluorescent intensity.

NaHS and DATS at the concentrations tested had no significant effect on PMNL phagocytosis. Neither the percentage of PMNLs taking up *E. coli* (Figure 2A,C) nor the number of fluorescent-labeled *E. coli* cells taken up per PMNL, as measured by mean fluorescent intensity (MFI), was affected (Figure 2B,D), except that a slight, non-significant increase was observed in the presence of NaHS.

### 2.3. Oxidative Burst

**Figure 3:**
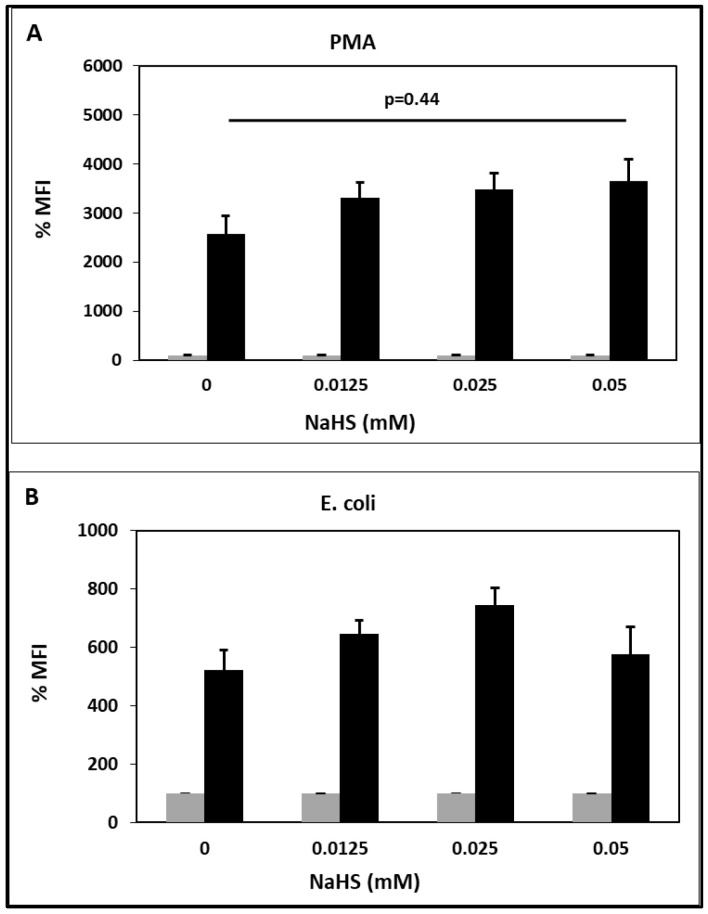
Effect of the H_2_S-releasing substance NaHS on PMNL oxidative burst in the absence (gray bars) or presence (black bars) of PMA (**A**) or *E. coli* (**B**). The MFI of the unstimulated sample in the absence of substance was set as 100%. n = 4 for 0.0125 and 0.025 mM; n = 6 for 0.05 mM. NaHS: sodium hydrosulfide, PMA: phorbol 12-myristate 13-acetate, MFI: mean fluorescent intensity.

NaHS did not affect the basal oxidative burst at all the tested concentrations (Figure 3). However, NaHS increased the phorbol 12-myristate 13-acetate (PMA)-stimulated oxidative burst in a concentration-dependent manner (Figure 3A). NaHS also increased the oxidative burst stimulated by *E. coli* (Figure 3B). However, this effect did not reach statistical significance. Therefore, NaHS primes PMNLs for the production of ROS activated by PMA or *E. coli*.

**Figure 4:**
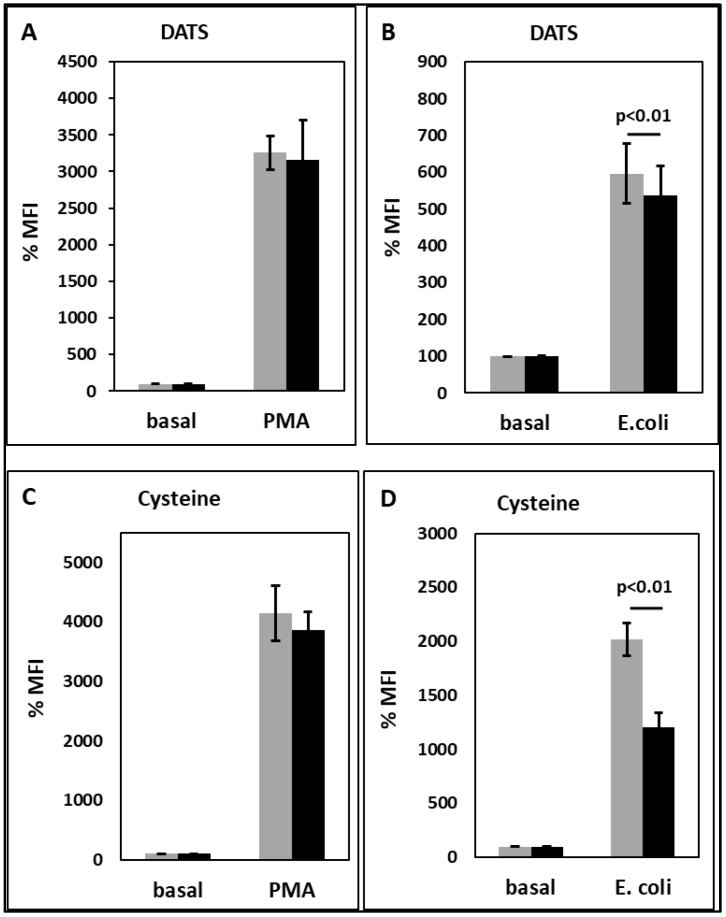
Effect of the H_2_S-releasing substances DATS at a concentration of 50 µM (**A**,**B**) and cysteine at a concentration of 20 mM (**C**,**D**) on PMNL oxidative burst in the absence (gray bars) or presence (black bars) of PMA (**A**,**C**) or *E. coli* (**B**,**D**). The MFI of the unstimulated sample in the absence of substance was set as 100%. n = 6 for A and B; n = 7 for C and D. DATS: diallyl trisulfide, PMA: phorbol 12-myristate 13-acetate, MFI: mean fluorescent intensity.

Neither DATS nor cysteine had an effect on the basal or PMA-stimulated oxidative burst (Figure 4A,C). However, both DATS and cysteine significantly decreased the oxidative burst stimulated by *E. coli* (Figure 4B,D).

### 2.4. Apoptosis

**Figure 5:**
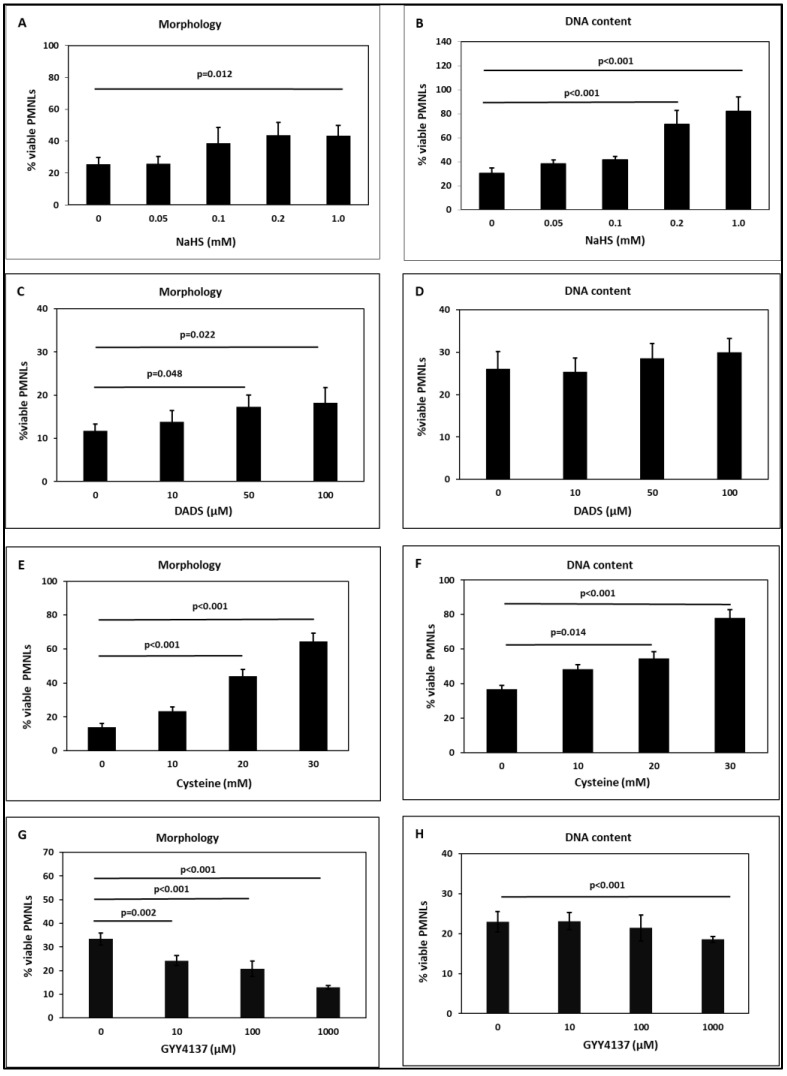
Effect of the H_2_S-releasing substances NaHS (**A**,**B**), DADS (**C**,**D**), cysteine (**E**,**F**), and GYY4137 (**G**,**H**) on PMNL apoptosis. Apoptosis was determined by assessing morphological features according to the fluorescence upon ethidium bromide uptake (**A**,**C**,**E**,**G**) and by measuring the DNA content (**B**,**D**,**F**,**H**). n = 3–5 for (**A**,**B**); n = 6 for (**C**–**F**); n = 8 for (**G**,**H**). NaHS: sodium hydrosulfide, DADS: diallyl disulphide.

NaHS, DADS, and cysteine increased the percentage of viable PMNLs, i.e., reduced PMNL apoptosis in a concentration-dependent manner (Figure 5A–F). This effect was observed both by evaluating morphological features (Figure 5A,C,E) and by the DNA content (Figure 5B,D,F). The percentage of viable PMNs was higher when measuring the DNA content as compared to the assessment of PMNL morphology, because DNA fragmentation is a later event during spontaneous PMNL apoptosis compared to morphological changes [20]. This could also explain the finding that no concentration-dependent effect of DADS on the DNA content was observed (Figure 5D). 

In contrast to NaHS, DADS, and cysteine, GYY4137 increased PMNL apoptosis and thereby decreased their viability concentration dependently (Figure 5G,H). These results suggest that, in the model system adopted here, GYY4137, although endowed with an H_2_S-releasing activity, may prevalently act through alternative pathway(s).

**Figure 6:**
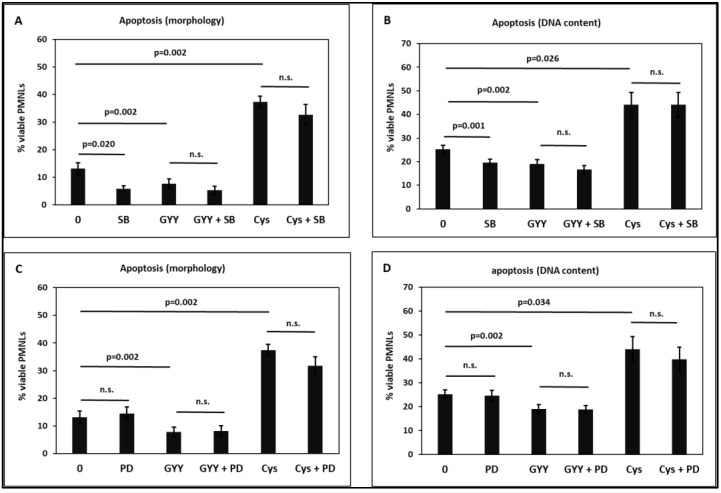
Effect of 1 mM GYY4137 (GYY) and 30 mM cysteine (Cys) on PMNL in the absence and presence of 30 µM SB203580 (SB; p38 MAPK inhibitor; (**A**,**B**)) and 50 µM PD98059 (PD; ERK inhibitor; (**C**,**D**)). Apoptosis was determined by assessing morphological features (**A**,**C**) and by measuring the DNA content (**B**,**D**). n = 8. MAPK: mitogen-activated protein kinase, ERK: extracellular signal-regulated kinase. n.s.: non-significant.

To elucidate the involvement of signal transduction pathways in the effect of the apoptosis-decreasing cysteine and the apoptosis-accelerating compound GYY4137, we used several specific signal transduction inhibitors. The p38 mitogen-activated protein kinase (MAPK) inhibitor SB203580 alone increased PMNL apoptosis (Figure 6A,B), consistent with reports that p38 MAPK is responsive as a part of a cell survival signaling pathway in PMNLs [22,23,24,25]. 

The p38 MAPK inhibitor did not significantly affect the attenuating effect of cysteine, suggesting that H_2_S synthesized from cysteine targets signaling downstream of p38. On the other hand, SB203580 had a slight additive effect on GYY4137-induced apoptosis. Additive effects point to different downstream signaling pathways [26,27,28], suggesting that p38 MAPK is not involved in GYY4137-triggered apoptosis.

The extracellular signal-regulated kinase (ERK) inhibitor PD98059 did not alter the apoptosis-modulating effects of GYY4137 and cysteine, and nor did it affect spontaneous PMNL apoptosis alone (Figure 6C,D). 

**Figure 7:**
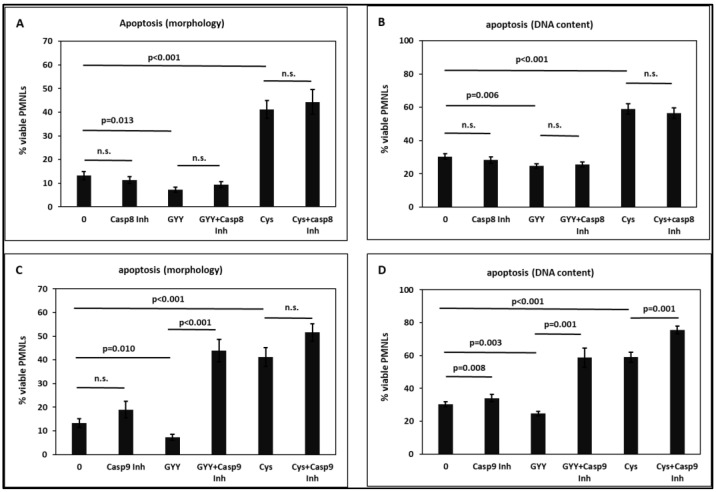
Effect of 1 mM GYY4137 (GYY) and 30 mM cysteine (Cys) on PMNL in the absence and presence of 20 µM Z-IETD-FMK (caspase 8 inhibitor; (**A**,**B**)) or Z-LEHD-FMK (caspase 9 inhibitor; (**C**,**D**)). Apoptosis was determined by assessing morphological features (**A**,**C**) and by measuring the DNA content (**B**,**D**). n = 8. n.s.: non-significant.

Whereas the caspase 8 inhibitor Z-IETD-FMK had no effect, the caspase 9 inhibitor Z-LEHD-FMK attenuated the spontaneous PMNL apoptosis (Figure 7), indicating an involvement of the intrinsic apoptotic pathway. The caspase 8 inhibitor exerted no statistically significant interference on the GYY4137 or the cysteine effects on apoptosis (Figure 7A,B).

The caspase 9 inhibitor abrogated the effect of GYY4137 (Figure 7C,D), suggesting that the intrinsic pathway of apoptosis is mainly involved in GYY4137-induced PMNL apoptosis. The caspase 9 inhibitor showed an effect additive to the cysteine effect, indicating that different signaling pathways are involved in the inhibitory effect.

**Figure 8:**
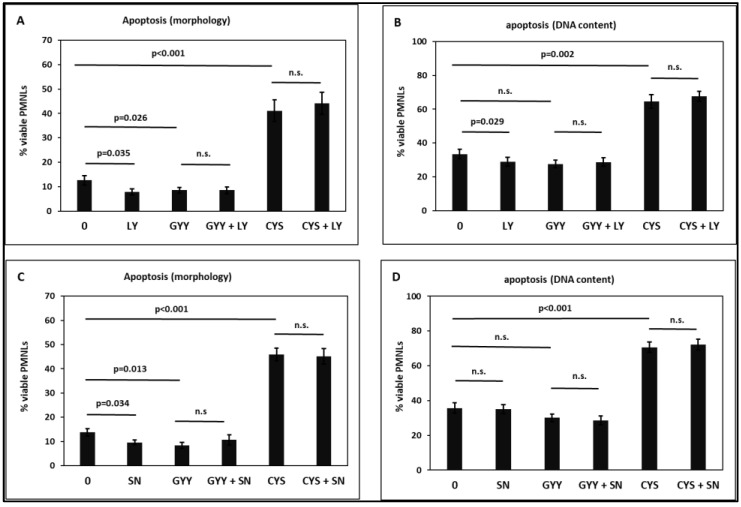
Effect of 1 mM GYY4137 (GYY) and 30 mM cysteine (Cys) on PMNL in the absence and presence of 25 µM LY294002 (PI3K inhibitor (**A**,**B**)) or 4 µM SN50 (NF-κB inhibitor (**C**,**D**)). Apoptosis was determined by assessing morphological features (**A**,**C**) and by measuring the DNA content (**B**,**D**). n = 8. PI3K: phosphoinositide 3-kinase, NFκB: nuclear factor kappa B. n.s.: non-significant.

The phosphoinositide 3-kinase (PI3K) inhibitor LY294002 decreased PMNL viability, i.e., accelerated PMNL apoptosis (Figure 8A,B), as described in the literature [29]. The PI3K inhibitor did not influence the effects of GYY4137 and cysteine, suggesting that both substances target signaling downstream of PI3K.

Consistent with the pro-survival, pro-inflammatory effect of nuclear factor kappa B (NF-κB), the inhibitor SN50 increased PMNL apoptosis. However, this effect was only observed when assessing morphological features (Figure 8C). As in the case of LY294002, SN50 did not affect the effects of GYY4137 and cysteine.

## 3. Discussion

**Figure 9:**
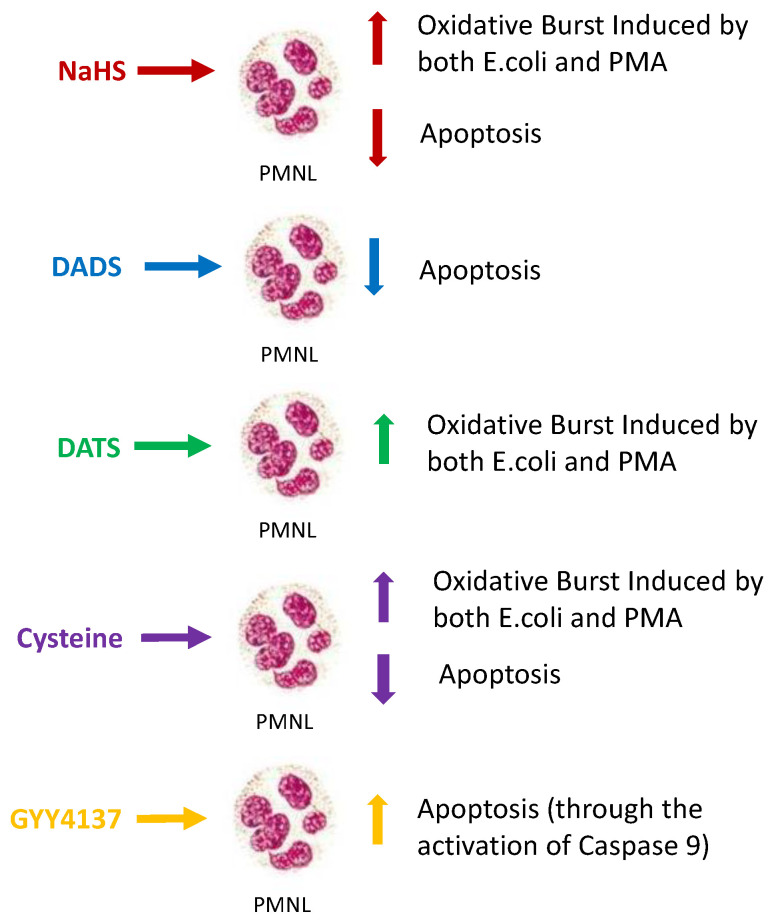
Schematic representation of the effects induced by the various hydrogen-sulfide-releasing compounds on PLMN functions.

In CKD patients, uremic toxins contribute to reduced levels of H_2_S, a substance that has a variety of beneficial effects in multiple biological systems. In this study we showed that essential functions of PMNLs, cells of the first-line non-specific immune defense and crucial for protection against bacterial and fungal infections, were modulated in vitro by H_2_S synthesized from several H_2_S-producing substrates and H_2_S-releasing precursors. (Figure 9). Depending on the concentration and the kinetics of the H_2_S release from the precursor compounds, both attenuating and stimulating effects on various functions of PMNLs were observed.

Along with the more familiar carbon monoxide and nitric oxide, H_2_S belongs to the family of gasotransmitters. H_2_S is largely dissociated (86%) under physiological conditions, and only 14% is present as a gas [1]. Because of its lipophilicity, H_2_S gas can easily diffuse through cell membranes independently from receptors and cause various local and systemic effects [30]. H_2_S is closely linked to several vascular diseases and is used as a new therapeutic target [31]. H_2_S is involved in the regulation of various physiological and pathological conditions in cardiovascular [32], renal [33], and central nervous systems [34]. H_2_S has a neuroprotective effect by increasing the production of the antioxidant glutathione in neurons exposed to oxidative stress [8,9]. In addition, H_2_S has effects on respiratory [35], reproductive [36], and digestive systems [37]. Implications of H_2_S production have also been reported in periodontitis development [38]. H_2_S decreases the production of pro-inflammatory cytokines, chemokines, and enzymes [4] and downregulates cAMP, a regulator of renin secretion from juxtaglomerular cells, thereby regulating blood pressure [39]. H_2_S synthesis is independent of O_2_. However, cystathionine beta-synthase (CBS), one of the H_2_S-producing enzymes, is a hemesensor protein [40]. By sensing hypoxia, it can block tubular transport and increase medullar blood pressure [41]. In endothelial cells, lanthionine (a prospective uremic toxin and a byproduct of H_2_S biosynthesis) hampers H_2_S release; reduces protein content and glutathionylation of the transsulfuration enzyme CBS; modifies the expression of miR-200c, miR-423, and vascular endothelial growth factor; and, most importantly in this context, increases intracellular Ca^2+^ levels [42]. Protein persulfidation, a posttranslational modification of cysteine residues to persulfides caused by H_2_S, is an important mechanism of H_2_S-mediated signaling pathways [43].

Divergent results have been reported regarding the effects of H_2_S on the immune system [44,45]. Some studies report a pro-inflammatory effect of H_2_S. In models of inflammation such as sepsis, endotoxic, and hemorrhagic shock, increased levels of H_2_S were observed. Furthermore, the cystathionine γ-lyase (CSE) inhibitor DL-propargylglycine leads to reduced inflammation [46,47,48,49,50]. Other studies described H_2_S as an anti-inflammatory molecule, e.g., inhibiting TNFα and lipopolysaccharides-stimulated NF-κB activation [51,52]. H_2_S modifies inflammatory responses on the level of endothelium and leukocytes [53]. In addition, it reduces monocyte cell adhesion and relevant inflammatory triggering by preventing ADAM17-dependent TNF-α activation [54].

The plasma levels of H_2_S in patients with coronary heart disease, hypertension, and smokers are lower than in the healthy reference group [5]. A study by Whiteman et al. showed that patients with obesity or type-2 diabetes had decreased H_2_S plasma levels [55]. CKD is associated with significantly reduced H_2_S production and H_2_S plasma levels [56]. In end-stage kidney disease patients, in contrast to the low levels of H_2_S, the plasma concentrations of cysteine and homocysteine are elevated [56]. Hyperhomocysteinemia is a well-known risk factor for cardiovascular disease and leads to downregulation of CBS, CSE, and 3-mercaptopyruvate sulfurtransferase (3-MST) in the kidneys and liver, resulting in decreased H_2_S plasma levels [57]. The deficiency of H_2_S and its anti-oxidant, anti-inflammatory, and cytoprotective properties may contribute to the progression of CKD and its mortality [2]. Infusion of L-cysteine into the renal artery to raise the endogenous production of H_2_S resulted in an increase in GFR [58]. 

The uremic milieu and uremic toxins in particular play a key role in the reduction of H_2_S levels in renal disease. Many risk factors affecting increased cardiovascular mortality in CKD are associated with uremic toxins and their derivatives [59]. The metabolism of sulfur-containing amino acids is mainly affected. Lanthionine, a by-product of H_2_S biosynthesis, has historically served as a marker for H_2_S production. Lanthionine plasma levels are considerably elevated in uremic patients, and it has been categorized as a new uremic toxin [14]. Lanthionine is removed to some extent by a single dialysis session, which is accompanied by an increase in H_2_S levels [13]. In endothelial cells, lanthionine inhibits H_2_S release by decreasing the protein content and glutathionylation of CBS. The uremic toxin nature of lanthionine has also been evaluated in a zebrafish animal model [60]. The uremic toxin indoxyl sulfate downregulates the H_2_S-generating enzymes CSE, CBS, and 3-MST [15]. This effect was reversed by inhibiting the receptor for indoxyl sulfate, the aryl hydrocarbon receptor. The uremic toxin cyanate is produced by the decomposition of urea. It can inhibit the scavenging of free radicals by H_2_S via carbamoylation and thereby contributes to the increased oxidative stress in CKD patients [19].

For H_2_S to serve as an endogenously produced messenger, tissue production and catabolism must result in intracellular microenvironments with a sufficiently high H_2_S concentration to activate a local signaling mechanism [61]. The concentration of H_2_S in mammalian plasma may be substantially increased in certain microenvironments, e.g., H_2_S release from bound sulfur [62]. Increased sulfide levels of up to 1 mM have been reported at infected sites in polymicrobial infections during periodontal disease [63].

H_2_S production from L-cysteine involves the enzymes CBS, CSE, and 3-MST. Pyridoxal phosphate is a coenzyme of CBS and CTH. Several H_2_S donors with potential therapeutic applications in cardiovascular diseases have been described [64,65,66]. In our in vitro assays, we used the H_2_S-producing substances NaHS, DATS and DADS, GYY4137, and cysteine. GYY4137 is a water-soluble synthetic H_2_S donor [67,68]. The main advantage of GYY4137 over NaHS is the significantly slower H_2_S-releasing process. Whereas H_2_S release by GYY4137 peaks after 6–10 min, the H_2_S release by NaHS peaks already within 5–8 s. This more gradual increase in concentration corresponds more closely to conditions in vivo [68]. Low H_2_S concentrations that persist for a relatively long period are more comparable to the physiological milieu in vivo and have no cytotoxic effect. On the other hand, high H_2_S concentrations peaking in a short time period correspond to pathological conditions that lead to the activation of specific signal pathways and thus to cell death [68]. DATS and especially DADS also release H_2_S at a lower rate than NaHS [69]. H_2_S production by DADS and DATS in tissue depends on glutathione. DADS–GTH interaction generates S-allyl-glutathione and allylperthiol, which induce H_2_S release through GTH [70]. 

In our chemotaxis assay, NaHS, DADS, and cysteine had no significant effect on PMNL migration (Figure 1). In septic mice, NaHS stimulated PMNL migration to the side of infection [71]. H_2_S-releasing substances increase the migration of PMNLs and work as anti-inflammators and anti-oxidants. Treatment of LPS-challenged naive mice with the H_2_S donor NaHS enhanced neutrophil migration [72]. H_2_S can also reduce leukocyte adherence, leukocyte infiltration, and edema formation [53].

NaHS and DATS had no significant effect on PMNL phagocytosis of *E. coli* bacteria (Figure 2). Cleasson et al. [73] reported that phagocytosis of *Streptococcus agalactiae* was only slightly affected by the presence of sulfide.

NaHS increased the PMA- and *E.coli*-stimulated oxidative burst. Therefore, NaHS primes PMNLs for the production of ROS activated by PMA and *E. coli* (Figure 3). Whereas priming of PMNLs is an important physiologic mechanism in regulating the immune defense [74], excessive PMNL priming results in inflammation and oxidative stress [75].

DATS and cysteine significantly decreased the oxidative burst of PMNLs stimulated by *E. coli* (Figure 4). The observation that both substances do not interfere with the direct activation of protein kinase C (PKC) by PMA but inhibit the receptor-mediated stimulation of the oxidative burst by *E. coli* suggests that H_2_S released by DATS and cysteine interferes with signaling upstream of PKC. Anti-oxidative and anti-inflammatory properties of DADS and DATS have been described. In epithelial cells, DADS reduces inflammation by inhibiting ROS production and NF-κB activation [76]. In a mice model of lipopolysaccharide (LPS)-induced acute lung injury, H_2_S released by the H_2_S donor GYY4137 led to reduced migration of PMNL and an impaired ROS production [77].

The synchronized elimination of activated PMNLs by apoptotic cell death is essential for the termination of inflammation [78]. A complex system of intracellular signaling pathways mediates PMNL death and is controlled by various extracellular stimuli, e.g., pro-inflammatory cytokines [79]. We found that NaHS, DADS, and cysteine reduced PMNL apoptosis, but GYY4137 had an apoptosis-enhancing effect (Figure 5). A difference in the kinetics of H_2_S formation might explain the differences in effects on apoptosis. Consistent with our results, Rinaldi et al. reported that H_2_S produced by NaHS attenuated PMNL apoptotic cell death and that this effect is due to inhibition of p38 MAPK and caspase 3 [80]. Shigemi et al. reported that DATS-mediated suppression of NF-κB signaling leads to induction of apoptosis [81]. However, the effect of allyl derivatives on apoptosis is controversial. Several studies found that DADS and DATS induce apoptosis [82,83,84,85]. On the other hand, studies on cardiomyocytes indicate an anti-apoptotic effect of DATS [86,87]. 

In agreement with the literature reporting that p38 MAPK mediates PMNL survival [22,23,24,25], we found that the p38 MAPK inhibitor SB203580 accelerated PMNL apoptosis (Figure 6A,B). The p38 MAPK inhibitor did not significantly affect the apoptosis-attenuating effect of cysteine, suggesting that H_2_S synthesized from cysteine targets signaling downstream of p38. SB203580 had an additive effect on GYY4137-induced apoptosis (Figure 6A,B). Additive effects indicate different downstream signaling [26,27,28,88,89], suggesting that GYY4137-triggered apoptosis is independent of the p38 MAPK pathway. Inhibition of ERK by PD98059 did not change PMNL apoptosis or the effects of GYY4137 and cysteine (Figure 6C,D). Results in the literature on the role of ERK in apoptotic pathways are not consistent, reporting both pro- and anti-apoptotic properties of ERK [90,91,92].

Whereas inhibition of caspase 8 had no effect on PMNL apoptosis, the caspase 9 inhibitor had an attenuating effect (Figure 7), showing a contribution of the intrinsic pathway. In a review article, Webb et al. described that spontaneous neutrophil apoptosis is independent of Fas ligation but is mediated by caspases 8 and 9 [93]. Caspase 9 inhibition abolished the GYY4137 effect (Figure 7C,D), indicating that the intrinsic apoptosis pathway is mostly involved in GYY4137-induced PMNL apoptosis. At the moment, we do not have a satisfactory explanation for the unexpected finding that the caspase 9 inhibitor showed a much stronger effect in the presence of GYY4137 than alone. Caspase 9 inhibition had an additive influence on the inhibitory effect of cysteine, suggesting different signaling pathways (Figure 7). 

The PI3K inhibitor LY294002 reduced PMNL viability (Figure 8A,B), consistent with the reported involvement of PI3K in anti-apoptotic signaling pathways [29]. Several growth-factor and survival-factor receptors activate PI3K [94,95]. Stimulated by second messengers, phosphoinositide-dependent protein kinase-1 phosphorylates protein kinase B, resulting in activation of NF-κB and transcription of anti-apoptotic genes [96]. The PI3K inhibitor did not affect either the apoptosis-enhancing effect of GYY4137 or the apoptosis-attenuating effect of cysteine (Figure 8), suggesting that both substances target signaling downstream of PI3K.

Consistent with the pro-survival, pro-inflammatory effect of NF-κB, we showed that the inhibitor SN50 increased PMNL apoptosis (Figure 8C,D). H_2_S is an endogenous inflammatory mediator by increasing the activity of NF-κB [48,53,97]. H_2_S produced by CSE stimulates DNA binding and gene activation of NF-κB. Its anti-apoptotic transcriptional activity is caused by sulfhydration of NF-κB [98].

A wide range of H_2_S in vivo concentrations has been reported in the literature: 8 µM [13], 38 μM [99], 34 μM [100], 46 μM [101], and 274 μM [102] have been measured. In in vitro experiments using H_2_S-donors, the volatility of H_2_S has to be considered. H_2_S volatilization is so fast that the applied H_2_S concentration is not reached. The loss of H_2_S produced from Na_2_S crystals is rapid and exponential, with half-times of 5 min [103,104]. In cumulative H_2_S dose–response studies on mouse aortas, only 9% of the expected value was measured [105]. Exogenous sulfide is rapidly removed from blood, plasma, or 5% bovine serum albumin in vitro. When sulfide was added to whole blood, the measured peak concentration typically did not exceed 20% of that added [106]. Sulfide consumption increased with increasing BSA concentrations [106]. Therefore, both time and protein concentration are of the greatest importance for the concentration of available H_2_S in biological samples [107].

It should be noted that samples were taken from healthy subjects for our study. We used whole blood for the phagocytosis and oxidative burst assays. As a result, we increased the current H_2_S concentration but observed effects without the interference by the uremic milieu. On the other hand, we used isolated PMNLs for the chemotaxis and apoptosis tests. Therefore, the cells were only exposed to the H_2_S released by the added substances. Furthermore, PMNLs from healthy subjects have not been exposed to uremic toxins, which pre-activate immune cells while impairing their ability to respond to stimuli. It has been shown that PMNLs from healthy subjects react differently to uremic toxins than PMNLs from CKD patients [108,109]. Mononuclear cells from CKD patients have a significantly decreased cytokine production when exposed to lipopolysaccharide [110] and a diminished proliferative response to antigens in vitro [111]. One limitation of this study is that we only determined the concentration of releasing substances based on the literature and did not measure the amount of H_2_S generated during experiments. 

## 4. Conclusions

We tested the effect of H_2_S-releasing substances on functions of PMNLs from healthy subjects in vitro. None of the tested substances had a significant effect on chemotaxis or phagocytosis under the experimental conditions. NaHS primed PMA- and *E. coli*-stimulated PMNL oxidative burst, whereas DATS and cysteine significantly reduced *E. coli-* stimulated oxidative burst but had no effect on PMA activation. NaHS, DADS, and cysteine reduced PMNL apoptosis, while GYY4137 had the opposite effect. Experiments with signal transduction inhibitor suggest that the intrinsic pathway of apoptosis is mainly involved in GYY4137-induced PMNL apoptosis. Investigating the effect of H_2_S on PMNLs from CKD patients and measuring the actual H_2_S concentration during the experiments are topics of future research projects.

## 5. Material and Methods

### 5.1. Healthy Subjects

We only included healthy subjects between 18 and 65 years of age. A total of 66 probands (35.5 ± 1.6 years; mean ± SEM) donated blood: 32 women (33.8 ± 2.1 years) and 34 men (37.1 ± 2.2 years). Some probands gave a blood sample several times, but only once per experimental set.

Excluded were people with signs of infection or inflammation, chronic diseases, or taking medications that influence the immune system. The blood donors suffered neither from renal dysfunction nor from any psychiatric or neurological disease.

### 5.2. Material

The following chemicals were purchased from Sigma-Aldrich (St. Louis, MO, USA): NaHS (catalog number #161527), DADS (#SMB00378), L-cysteine (#168149), GYY4137 (#SML0100), fMLP (#F3506), RNAse (#R4875), propidium iodide (#P4170), SB203580 (#S8307), PD98059 (#P215), LY294002 (#L9908), and SN50 (#SML1471). DATS was purchased from Cayman (Ann Arbor, MI, USA; #10012577). The caspase inhibitors were purchased from R&D Systems (Minneapolis, MN, USA): caspase 8 inhibitor Z-IETD-FMK (#FMK007) and caspase 9 inhibitor Z-LEHD-FMK (#FMK008).

### 5.3. Isolation of PMNLs

Discontinuous Ficoll-Hypaque (GE Healthcare Bio- Sciences AB, Uppsala, Sweden; #17144003) density-gradient centrifugation was used for the isolation of PMNLs. Ten mL of whole venous blood from healthy donors was collected in a sterile lithium heparin vacutainer tube (Greiner Bio-One GmbH, Kremsmünster, Austria) and then underlayered with 12 mL of Ficoll-Hypaque. After centrifugation, hypotonic lysis on ice with ammonium chloride buffer (157 mM NH_4_Cl, 10 mM KHCO_3_, and 0.1 mM ethylenediaminetetraacetate Na_2_) removed the erythrocytes. Then the PMNLs were collected by centrifugation at 4 °C and washed once with lysis buffer and twice with phosphate-buffered saline (PBS, pH 7.4; BioWhittaker Lonza Services, Verviers, Belgium; #BE17-513F)). The viability of the PMNL isolated by this protocol was >95%, as determined by the exclusion of ethidium bromide (GibcoBRL Life Technologies, Gaithersburg, MD, USA; #15585011) under the fluorescence microscope.

In preliminary experiments, we used one concentration of the H_2_S donor. Subsequently, we tested the concentration dependence of the observed effect. Therefore, different numbers of experiments were performed for the different concentration.

### 5.4. Chemotaxis

The chemotactic movement of PMNLs was measured by the under-agarose method as previously described [103]. Isolated PMNLs were suspended at a concentration of 0.5 × 10^6^/10 μL in PBS or in PBS containing the H_2_S-releasing substance. N-formyl-methionyl-leucyl-phenylalanine (fMLP) was used as a chemoattractant at a final concentration of 4.2 × 10^−7^ M. The plates were incubated for about 2 h at 37 °C, 5% CO_2_. After fixation of the PMNLs with methanol and paraformaldehyde (Fluka, Sigma-Aldrich Chemie GmbH, Buchs, Switzerland; #76240) and staining with Giemsa (Merck, Darmstadt, Germany; #1.09204.0500), the distance the cells migrated under the agarose was measured under the microscope.

### 5.5. Phagocytosis

PMNL phagocytosis was analyzed by flow cytometry in heparinized whole blood. The percentage of PMNL that had taken up fluorescein (FITC)-labeled opsonized *E. coli* and the amount of ingested *E. coli* per PMNL were determined using the “PhagoTest” kit (Celonic, Heidelberg, Germany; #10-0100). Sample histograms are shown in the Supplementary Figure S1.

### 5.6. Oxidative Burst

The oxidative burst of PMNLs was assessed in heparinized whole blood using Bursttest (Celonic, Heidelberg, Germany; #10-0200). Unlabeled opsonized *E. coli*, PMA, and the chemotactic peptide fMLP were used as stimulants, and dihydrorhodamine (DHR) 123 was used as fluorogenic substrate. The MFI was measured by flow cytometry on a BD FACSCanto II (BD Biosciences, San Jose, CA, USA). Sample histograms are shown in the Supplementary Figure S2.

### 5.7. Apoptosis

#### 5.7.1. Incubations

The spontaneous PMNL apoptosis was assessed as previously described [104]. PMNLs were isolated under sterile conditions and incubated at 6 × 10^6^ cells/mL for 20 h at 37 °C in PBS containing 100 U/mL penicillin–streptomycin (Gibco—Thermo Fisher Scientific, Waltham, MA, USA; #15140122). 

#### 5.7.2. Morphological Features

The fluorescent DNA-binding dyes ethidium bromide and acridine orange (Merck, #15931) were added to the PMNL suspension at a final concentration of 5 µg/mL each. DNA in apoptotic cells is condensed, while DNA of non-apoptotic cells is structured. Acridine orange bound to DNA appears green. Ethidium bromide is taken up by cells with a damaged plasma membrane and stains the DNA more intensely orange. As a result, viable non-apoptotic (green, structured nucleus), apoptotic (green, condensed nucleus), and secondary necrotic (orange, condensed nucleus) cells can be counted under the fluorescence microscope. 

#### 5.7.3. Analysis of the DNA Content by Flow Cytometry

Apoptotic cells have a lower DNA content due to DNA cleavage by activated nucleases. PMNLs were centrifuged at 360× *g* for 10 min and washed with PBS. Then 250 µL ice-cold 70% ethanol was added to the cell pellet and incubated on ice for 60 min. After centrifugation and washing with PBS, PMNLs were suspended in 200 µL PBS containing 250 µg/mL RNAse (type I-A) and 50 µg/mL propidium iodide. The samples were stored on ice, protected from light, until analyzed by flow cytometry analysis.

#### 5.7.4. Data Presentation

The data are presented as a percentage of viable PMNLs. Apoptotic PMNLs are in a stage between viability and secondary necrosis. Under in vivo conditions, apoptotic PMNLs would be readily phagocytosed. Therefore, viable PMNLs are most important for the interpretation of the results.

### 5.8. H_2_S-Releasing Substances

The concentration of H_2_S-releasing substance has been chosen based on the available values in literature (Table 1).

### 5.9. Statistics

Data were checked for normality with QQ-plots. In case non-normality was detected, we used the Wilcoxon test to compare groups. For more than two groups, a Kruskal–Wallis test followed by Dunn’s test was used to analyze the differences. In cases where normality could be assumed, a mixed model was used. For multiple pairwise comparisons and comparison to a control group, the Bonferoni–Holm method and the Dunnett test were applied, respectively. Data are presented as mean values ± standard error of the mean (SEM). A *p*-value less than 0.05 was considered significant. Statistical analysis was performed with SAS 9.4 for Windows (Cary, NC, USA) and R version 4.0.4 with the package FSA [131,132].

## Figures and Tables

**Table 1:** H_2_S-releasing substances.

Substance	Concentration	Cells	Effect	Comment	Ref.
NaHS	0.2–0.4 mM	Lymphocytes (CD8+, NK)	Apoptosis ↑	Caspase independent, glutathione dependent	[112]
NaHS	10–75 µM	Lung fibroblasts	Apoptosis ↑	12–48 h	[113]
NaHS	10, 100, 300 µM	Neutrophils (mice)	(MIP-2 induced) chemotaxis ↑		[72]
NaHS	10 mM	Jurkat	Apoptosis ↑ (caspase 9)	15 min	[114]
NaHS	10–1000 µM	HUVEC 3T3 fibroblasts	(IRI-induced) apoptosis ↓		[115]
NaHS	0.23–3.66 mM	Neutrophils	Apoptosis ↓		[80]
NaHS	100, 500, 1000 µM	Endothelial cells	Apoptosis ↑	“100 µM safe in vitro concentration”	[54]
NaHS	100, 200 µM	RAW 264.7	LPS-stimulated PGE_2_- biosynthesis ↓	↑ (n.s.) at 1000 µM, “biphasic effect”	[52]
Na_2_S	100 µM	SMC	Ca^2+^ release ↓		[116]
Na_2_S	25 µM	Neutrophils	NETosis	PMA-induced	[117]
DATS	100, 200 µM	HepG2	H_2_O_2_ ↑	not for DADS	[118]
DATS	100 µM	HepG2	Caspase 3 ↑	not for DADS	[118]
DATS	20 µM	U937	Apoptosis ↑	not in THP-1, HL-60, K562 cells	[119]
DATS	1–10 µM	Cardiomyocytes	Glucose-induced apoptosis ↓ ROS ↓	NFκB ↓	[87]
DATS	100 µM	Pancreatic cancer cells	Apoptosis ↑		[120]
DATS	5, 10 µM	Cardiomyocytes	Glucose-induced apoptosis ↓		[86]
DATS	1–5 µM	WEHI-3	Apoptosis ↑		[121]
DATS	12, 24 µM	Rat hepatic stellate cells	Apoptosis ↑		[122]
DATS	10–100 µm	“several human cancer cells”	Apoptosis ↑		[122]
DATS	20, 40 µM (5–120 µM)	Human osteosarcoma cells	Apoptosis ↑ ROS ↑		[82]
DATS	0–50 µM	Primary effusion lymphoma cells	Apoptosis ↑		[81]
DATS	1–5 µM	Ethanol-stimulated L02	Apoptosis ↓ ROS ↓		[123]
DADS	5–50 µM	Lung fibroblasts	Proliferation ↓		[124]
DADS	5–300 µM	KG1α	Apoptosis ↑	24, 48, 72 h	[84]
DADS	1–100 µM	C28I2 Chondrocytes	(IL1β-induced) ROS ↓ (mitochondrial) apoptosis ↓	2–24 h 25 µM after 24 h not cytotoxic	[125]
DADS	1, 5, 10 µg/ml	BAR-T cells	Apoptosis ↑ (DCA-induced) ROS ↓	1, 3, 6, 12 h	[76]
DADS	25–250 µM	A549	Apoptosis ↑ ROS ↑		[126]
DADS	10, 20 mg/l	HL-60	Apoptosis ↑ ROS ↑		[85]
Cysteine	10 mM	SMC	Ca^2+^ release ↓		[116]
Cysteine	10–1000 µM	Neutrophils	[Ca^2+^]_i_ ↑		[127]
GYY4137	1 mM	Hoxb8 neutrophils	ROS ↓ Endothelial transmigration ↓		[77]
GYY4137	200 µM	Neutrophils	(LPS stim.) ROS ↓ (LPS inhibited) apoptosis ↑		[128]
GYY4137	500 µM	RAW264.7cells	NF-κB activation (LPS) ↓		[129]
GYY4137	400 µM	MCF-7	Apoptosis	Not for IMR90 cells	[130]
GYY4137	100–1000 µM	RAW 264.7	LPS-stimulated PGE_2_-, NO_2_^−^-, TNFα-, IL-1β- biosynthesis ↓		[52]

HepG2: human hepatoblastoma cells. U937: human leukemia cells. WEHI-3: murine leukemia cells. L-02: human fetal hepatocyte line. KG1α: leukemia cell line. BAR-T cells: Barrett’s carcinoma precursor epithelial cells. A549: lung cancer cells. Hoxb8 neutrophils: conditionally HoxB8-immortalized mouse hematopoietic progenitors are suitable for in vitro differentiation of a range of myeloid cells, including neutrophils. RAW264.7cells: monocyte/macrophage-like cells, originating from Abelson leukemia virus transformed cell line derived from BALB/c mice. MCF-7 cells: epithelial cell line isolated from the breast tissue of a patient with metastatic adenocarcinoma. IMR90 cells: normal human lung fibroblasts. RAW 264.7: murine macrophages. IRI: Ischemia-reperfusion injury. The arrows ↑ and ↓ indicate increase and decrease, respectively, of the indicated parameter. n.s.: non-significant.

## Data Availability

The data presented in this study are available on request from the author.

## Supplementary Materials

The following supporting information can be downloaded at: https://www.mdpi.com/article/10.3390/toxins15030198/s1, Figure S1: Sample histograms PhagoTest; Figure S2: Sample histograms BurstTest.
