# Supplementary material for: Effect of Hydrogen Sulfide on Essential Functions of Polymorphonuclear Leukocytes
Source: Toxins (Basel). 2023 Mar 4;15(3):198. doi: 10.3390/toxins15030198 (PMC10058000; doi:10.3390/toxins15030198)
Supplement: Supplementary file 1 [file toxins-15-00198-s001.zip › toxins-2198888-supplementary.pdf]

Figure S1. Sample histograms PhagoTest

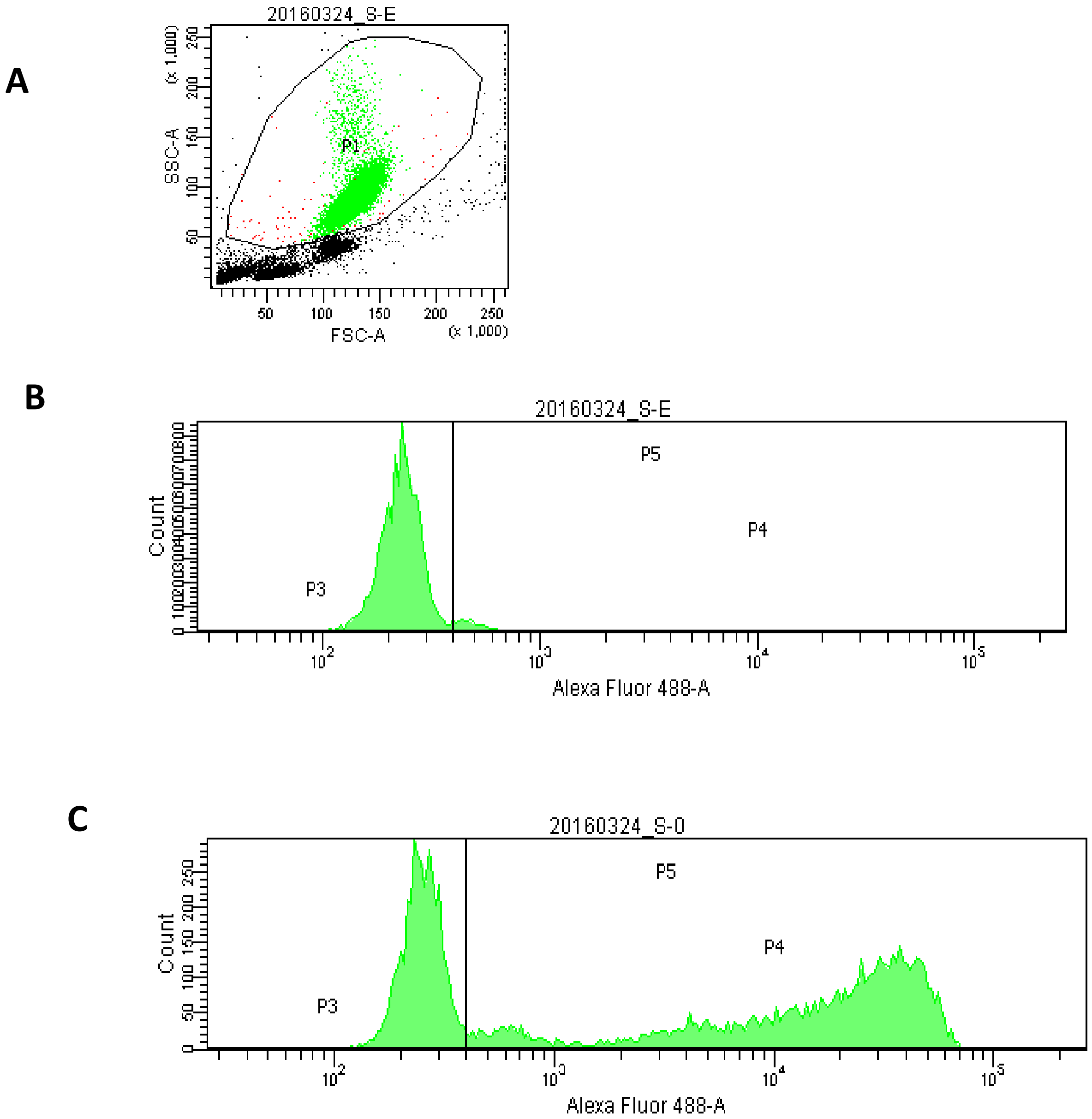

A: Forward FSC)/ and sideward (SSC) scatter histogram to gate the PMNL population (P1) in whole blood. **B and C:** Fluorescence intensity of PMNLs phagocytosing fluorescence-labeled E. coli before (**B**) and after (**C**) incubation. The percentage of phagocytosing PMNLs is calculated from the number of positive cells (P4) and of all counted cells (P5). The mean fluorescence intensity (MFI) of all counted cells (P5) serves as a measure for the number of E. coli taken up per PMNL.

Figure S2. Sample histograms BurstTest

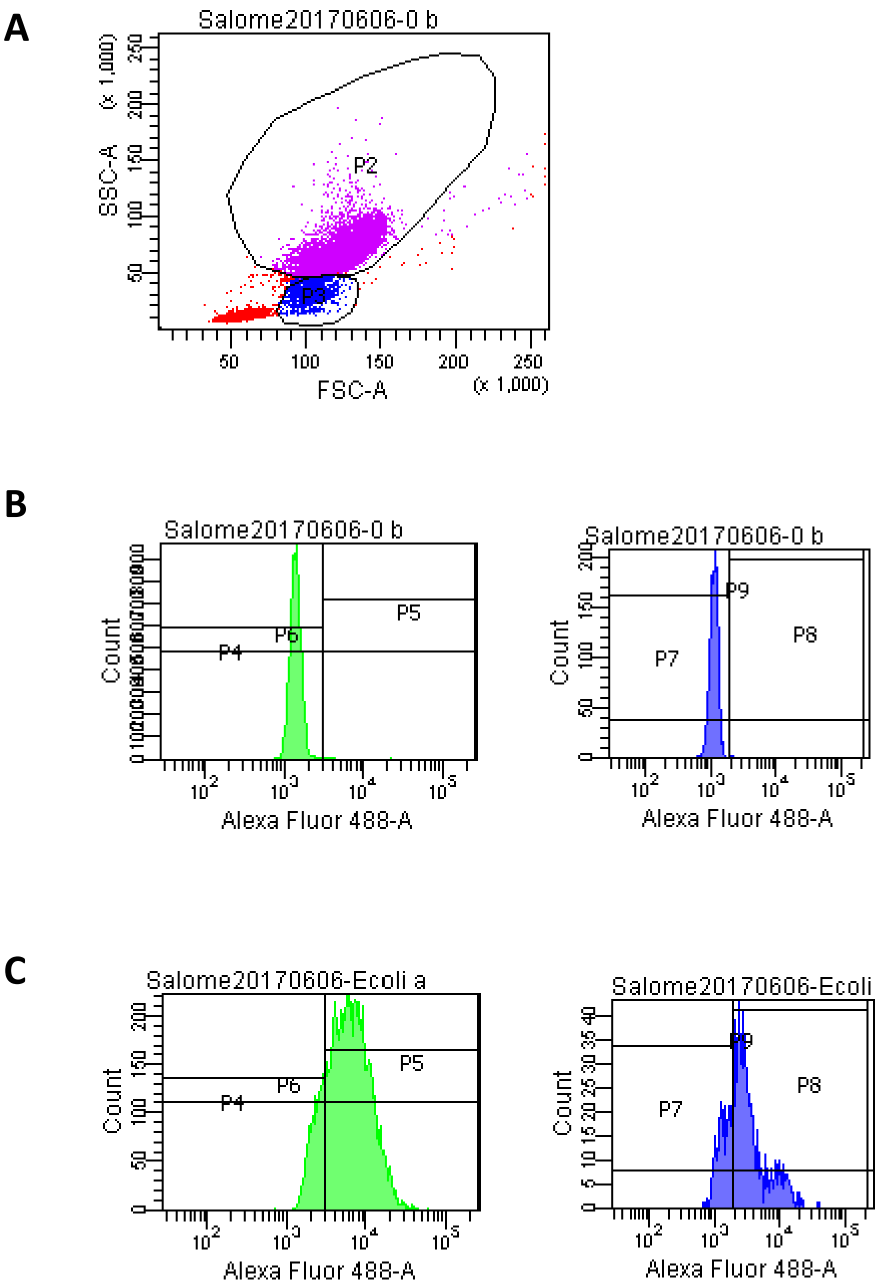

A: Forward FSC)/ and sideward (SSC) scatter histogram to gate the PMNL population (P2) in whole blood. The evaluation of the monocyte data is not part of this publication. **B and C:** The mean fluorescence intensity (MFI) of PMNLs (left histograms, green; P4) is taken as a measure of the oxidative burst activity. Unstimulated (**B**) and stimulated (in this example by E. coli; **C**) cells.
